# Evaluating the impact of Affordable Care Act repeal on America's opioid epidemic

**DOI:** 10.1371/journal.pmed.1002380

**Published:** 2017-08-29

**Authors:** Leana S. Wen, Evan B. Behrle, Alexander C. Tsai

**Affiliations:** 1 Baltimore City Health Department, Baltimore, Maryland, United States of America; 2 Chester M Pierce, MD Division of Global Psychiatry, Massachusetts General Hospital, Boston, Massachusetts, United States of America; 3 Harvard Center for Population and Development Studies, Cambridge, Massachusetts, United States of America

## Abstract

In this month’s Editorial, Health Commissioner of the City of Baltimore Leana S. Wen and co-authors discuss why the United States needs Medicaid to address its epidemic of opioid abuse.

*A previous version of this Editorial appeared July 27*, *2017 on PLOS’* Speaking of Medicine *blog*.

The November 2016 United States elections resulted in a Republican sweep of the presidency and both chambers of Congress. Republicans’ first major policy priority has been to “repeal and replace” the Obama administration’s effort to reform healthcare, the Patient Protection and Affordable Care Act (ACA), signed into law in 2010. While the repeal process faltered in the Senate, suffering a major legislative defeat in late July 2017, its resurrection remains a possibility. To date, a key component of proposed legislation from both the House and Senate has been severe cuts to Medicaid, which currently provides the lion’s share of health insurance for low-income Americans.

These legislative proposals have been introduced at a time when the U.S. is experiencing an epidemic of opioid addiction and overdose. In 2015, there were more than 2.6 million Americans with opioid use disorder (OUD) [1]. During the same year, more than 33,000 Americans died of overdoses involving one or more opioids, corresponding to an age-adjusted opioid-related death rate of 10.4 per 100,000 [2]—more than triple the rate in 2000 [3]. The U.S. now accounts for about a quarter of the world’s drug-related deaths [4].

The tragedy of opioid overdose is compounded by the fact that evidence-based treatments have existed for more than 4 decades. The acute phase effects of opioid overdose can be blocked through the administration of naloxone, a U.S. Food and Drug Administration (FDA)-approved medication available at many pharmacies. In the chronic phase, persons with the disease of OUD can be successfully treated and enter long-term recovery through a combination of medication-assisted treatment (MAT) and psychosocial support. The FDA has approved three medications for MAT—methadone, buprenorphine, and naltrexone—all of which are currently available in generic form (with the exception of extended-release naltrexone). Notwithstanding rhetoric about “replacing one drug with another”—or more legitimate concerns about adverse effects like respiratory depression—evidence for the efficacy of MAT prescribed in combination with psychosocial support in the treatment of OUD is robust: it suppresses illicit drug use more effectively than placebo and other treatments that do not use MAT [5–6], and it reduces criminal behavior [7] and both all-cause and overdose mortality [8].

Despite this evidence, only 1 in 10 Americans with substance use disorders receive treatment [1]. Nearly one-third of all those who did not seek treatment cite cost or lack of insurance coverage as a reason [1]. The treatment gap represents a substantial inefficiency for American taxpayers given that treatment can pay for itself by averting the medical morbidity and mortality—including HIV infection, overdoses, and hepatitis C—services utilization, and criminality associated with substance abuse [9]. According to the National Institutes of Health, every $1 invested in addiction treatment saves society $12 [10].

Medicaid cuts like the kind proposed in Congress [11–13] would make it significantly harder for those with OUD to access treatment. Nearly one-third of all those who receive substance use disorder treatment rely on Medicaid [1], and the program’s support for OUD treatment has grown rapidly in recent years, with growth concentrated in states that participated in the ACA’s expansion of Medicaid [14].

The importance of Medicaid in the treatment of OUD cannot be overstated for cities like Baltimore, where it provides coverage to one-third of all residents [15]. In Baltimore, Medicaid enrollees can purchase two doses of naloxone for $1—a potentially lifesaving discount compared with the $100-$4,500 sticker price [16]. A 2015 blanket prescription for naloxone issued by the first author (LSW), empowered by legislation passed by the Maryland legislature, enabled every Baltimore resident to receive training and obtain naloxone at any pharmacy. Between 2015 and 2017, approximately 1,000 lives were saved in Baltimore by lay people administering naloxone [17]. Other major efforts to combat the opioid epidemic in Baltimore would be severely compromised if Medicaid were cut, including programs to expand access to MAT and to construct a behavioral health crisis center to stabilize patients and connect them with care.

Plans to repeal and replace the ACA have threatened access to OUD treatment in another way: they would weaken or even eliminate the requirement that marketplace plans cover “essential health benefits,” which currently require insurance companies to cover OUD treatment. This means that, in addition to the millions of Americans who would lose their health insurance coverage entirely, there would also be many more **insured** Americans whose insurance would no longer cover OUD treatment [18]. The plans would also have allowed states to waive the requirement to cover preexisting conditions, immediately pricing people with OUD out of the individual market.

One version of the Senate bill attempted to ameliorate the effects of the Medicaid cuts by including $45 billion set aside for OUD treatment. This amount, however, would not adequately compensate for the reduction in future outlays associated with the loss of Medicaid and marketplace coverage: one estimate places the cost of treating OUD and its common comorbidities among Americans at or below 200% of the federal poverty line at $183 billion over the next 10 years [19].

Based on our reading of the evidence, Medicaid cuts of the kind proposed in House and Senate bills would have devastating consequences for the millions of Americans suffering from OUD. Patients who lose health insurance coverage and the ability to pay for their treatment may go into withdrawal and see no other choice but to turn to illicit opioids—with overdose and death as the possible result.

The consequences of untreated addiction extend beyond individual patients. Pregnant women with OUD are at risk for giving birth to babies with neonatal abstinence syndrome [20–21]. Children who grow up in homes affected by substance abuse are much more likely to suffer from OUD themselves as adults [22]. Parental incarceration for OUD-associated criminal activity also has deleterious intergenerational health and economic consequences [23–24].

No matter what, the American people will bear the cost of this epidemic—either by paying for treatment now or by paying for the medical, economic, and social consequences of denying it later. The choice should be clear.

## Abbreviations

ACA: the Patient Protection and Affordable Care Act
FDA: Food and Drug Administration
MAT: medication-assisted treatment
OUD: opioid use disorder
